# *Rickettsia africae* Infection in Man after Travel to Ethiopia

**DOI:** 10.3201/eid1511.090521

**Published:** 2009-11

**Authors:** Dorothea Stephany, Pierre Buffet, Jean-Marc Rolain, Didier Raoult, Paul H. Consigny

**Affiliations:** Institut Pasteur, Paris, France (D. Stephany, P. Buffet, P.H. Consigny); Université de la Méditerranée, Marseille, France (J.M. Rolain, D. Raoult).

**Keywords:** Rickettsia africae, African tick-bite fever, travel, Ethiopia, France, letter

**To the Editor:** The first human case of African tick-bite fever was described in 1992 as occurring in Zimbabwe. The causative agent was identified as a new serotype of the spotted fever group (SFG) rickettsiae and named *Rickettsia africae* (*1*). These findings confirmed observations made by Pijper in the 1930s, which suggested that there were 2 different kinds of human SFG rickettsioses in sub-Saharan Africa: Mediterranean spotted fever caused by *R*. *conorii* and transmitted by *Rhipicephalus* species, ticks of dogs, and African tick-bite fever caused by *R*. *africae* and transmitted by *Amblyomma* species, ticks of cattle and wild ungulates. African tick-bite fever has subsequently been diagnosed in patients from several other sub-Saharan countries and also from the West Indies (*2*,*3*).

In a recent analysis of the spectrum of diseases among returning travelers, tick-borne spotted fever was (after malaria) the second most frequent cause of systemic febrile illness among those returning from sub-Saharan Africa. It occurred more frequently than typhoid fever and dengue fever (*4*). The following case description reports an infection with *R*. *africae* in a man in France who recently returned from Ethiopia.

On November 4, 2005, a 62-year-old French man sought care at the Medical Center of the Institut Pasteur in Paris for fever, along with chills, headache, neck and shoulder pain, and fatigue over the previous 4 days. At the onset of these symptoms he had noticed dark nodular lesions on his neck and his left groin followed 2 days later by a slightly painful eruption on his arms and his trunk. He had spent a month in southwest Ethiopia, north of Kelem near the Sudanese border, and returned to France on October 26, 2005. While in Ethiopia, he had assisted with a production of a documentary film about an Ethiopian tribe and had been in contact with cattle in the villages. He had not noticed any tick bites. On physical examination he had a fever of 38°C, a nodular lesion with a central dark crust on his neck, a second lesion on his left inguinal fold (Figure, panel A), and a vesicular eruption on his arms and his trunk (Figure, panel B). Leukocyte count was 3,200, including 1,869 neutrophils and 867 lymphocytes. The platelet level was 174,000/mm^3^. The C-reactive protein level was 28.3 mg/L. The aspartate aminotransferase level was slightly elevated. The patient was treated with doxycycline 200 mg/day for 1 week for suspected African tick-bite fever. Follow-up showed a quick recovery from his symptoms except for fatigue that persisted for ≈1 month.

**Figure:**
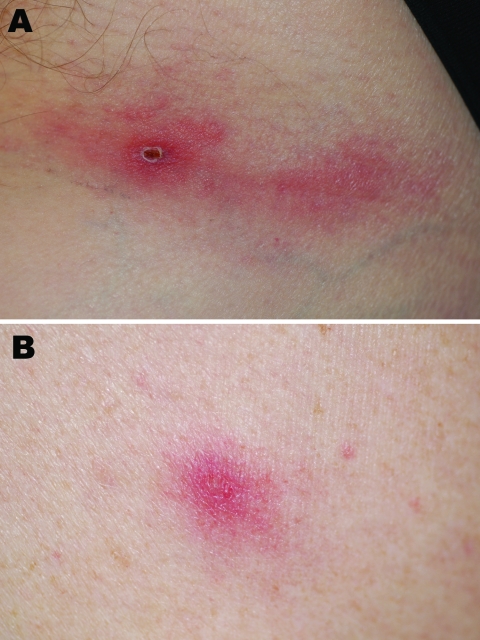
Inoculation eschar on left inguinal fold (A) and vesicular skin lesion (B) in a traveler recently returned to France from Ethiopia.

A commercial immunofluorescence assay for *R*. *conorii* and *R*. *typhi* immunoglobulin G performed both on an initial blood sample and a second sample taken 1 week later were negative. A blood sample and a biopsy specimen of the inguinal eschar were sent to the National Reference Center of Rickettsiae in Marseille, France. Although cellular culture of both specimens and molecular testing of the blood sample were negative, PCR for the sequences of citrate synthase (GenBank accession no. RAU59733, 93.1% homology) and rickettsial OmpA (GenBank accession no. RAU83436, 99.3% homology) applied on the skin biopsy detected *R*. *africae* and confirmed the diagnosis of African tick-bite fever.

From 1969 to 1971, SFG rickettsiae were isolated from *Amblyomma* spp. ticks collected in Ethiopia. They were regarded as *R*. *conorii* or as closely related bacteria (*5*). Later, more specific tests using western immunoblots with monoclonal antibodies showed that these rickettsiae differed from *R*. *conorii* (*6*). In 1992 SFG rickettsiae isolated from *Amblyomma* ticks collected in Zimbabwe and from the blood of a patient in Zimbabwe were compared to *R*. *conorii*, to other pathogenic SFG rickettsiae, and to a SFG rickettsia isolated from an *Amblyomma* spp. tick in Ethiopia 20 years before. The SFG rickettsia isolates from Ethiopia were identical to isolates obtained in Zimbabwe from the *Amblyomma* ticks and the patient’s blood and were different from *R*. *conorii* and other pathogenic SFG rickettsiae. This new serotype of SFG rickettsiae was named *R*. *africae* (*1*,*7*). A recent study confirmed the presence of *R*. *africae* in ticks collected in Ethiopia, as well as *R*. *aeschlimanii* (*8*). Thus, evidence of *R*. *africae* in Ethiopia has been known for a long time.

The geographic distribution of African tick-bite fever is related to the presence of *Amblyomma* spp. ticks, vectors and reservoirs of *R*. *africae*. Consequently African tick-bite fever should also be considered as a possible diagnosis in patients with febrile illness returning from countries where *R*. *africae* has been detected in *Amblyomma* ticks, even if a human infection has not yet been reported (*9*,*10*).
